# Advancements in Therapy for Acute Lymphoblastic Leukemia: Blinatumomab

**DOI:** 10.6004/jadpro.2016.7.1.6

**Published:** 2016-01-01

**Authors:** Lindsay Hladnik,1, Kristan Augustin,2, Sean DeFrates,3

**Affiliations:** 1Washington University Medical School, St. Louis, Missouri; 2Barnes-Jewish Hospital, St. Louis, Missouri; 3Northwestern Memorial Hospital, Chicago, Illinois

Acute lymphoblastic leukemia (ALL) is a rare form of leukemia, with an estimated 6,250 new cases diagnosed in the United States in 2015 and approximately 1,450 deaths (American Cancer Society, 2015). With current available induction therapies, complete remission (CR) rates in adults are approximately 75% to 90% (Faderl et al., 2010; Bassan & Hoelzer, 2011). After induction therapy, patients with ALL may then receive intensification, consolidation, and maintenance courses of chemotherapy or allogeneic hematopoietic stem cell transplant (HSCT). Although CR is obtained in the majority of patients with ALL, maintaining a durable CR is challenging despite the numerous phases of therapy. Standard chemotherapy regimens have resulted in an average cure rate of only 35% (Bassan & Hoelzer, 2011).

There are limited treatment options for patients with relapsed or refractory Philadelphia chromosome–negative (Ph–) ALL. Options may include enrollment into clinical trials or single- or multiagent chemotherapy regimens. Historically, these patients have had very poor outcomes, with reported CR rates of approximately 20% to 30% and a median overall survival (OS) of 3 to 6 months (National Comprehensive Cancer Network [NCCN], 2014; Topp et al., 2015). Given the poor outcomes seen with chemotherapy regimens in this setting, novel agents, such as blinatumomab (Blincyto), with alternative mechanisms of action are needed.

## MECHANISM OF ACTION

Blinatumomab is a first-in-class immunotherapy agent called a bispecific T-cell–engager (BiTE) antibody. The two antigens targeted by the bispecific design include CD3, which is found on cytotoxic T cells, and CD19, which is found on B lymphocytes throughout their development. The surface antigen CD19 is stably expressed on the majority of B-cell ALL blasts, making it a useful target for immunotherapy. Simultaneous binding of the CD3-positive T cells and CD19-positive B lymphocytes by blinatumomab leads to T-cell–mediated lysis of the CD19-positive normal and malignant B cells. In essence, blinatumomab assists the patient’s immune system by bringing the cancerous cells and the cytotoxic cells in proximity, so T-cell–mediated lysis of the malignant B cells can occur (Topp et al., 2011, 2015).

## CLINICAL TRIALS/EFFICACY

The first confirmed responses of blinatumomab were observed in a phase I dose escalation study including 38 patients with non-Hodgkin lymphoma (NHL; Bargou et al., 2008). Patients received 0.0005 to 0.06 mg/m²/day as a continuous infusion for 4 weeks every 6 weeks for up to 3 cycles. All responses occurred at a dose of at least 0.015 mg/m²/day, with an overall response rate (ORR) of 28.9%. Since then, blinatumomab has been studied in a variety of patient populations, including adults with relapsed/refractory Ph– and Ph+ B-cell precursor ALL (B-ALL), elderly patients with newly diagnosed B-ALL, pediatric and adolescent patients with relapsed/refractory B-ALL, and those with relapsed/refractory NHL (Table 1).

**Table 1:**
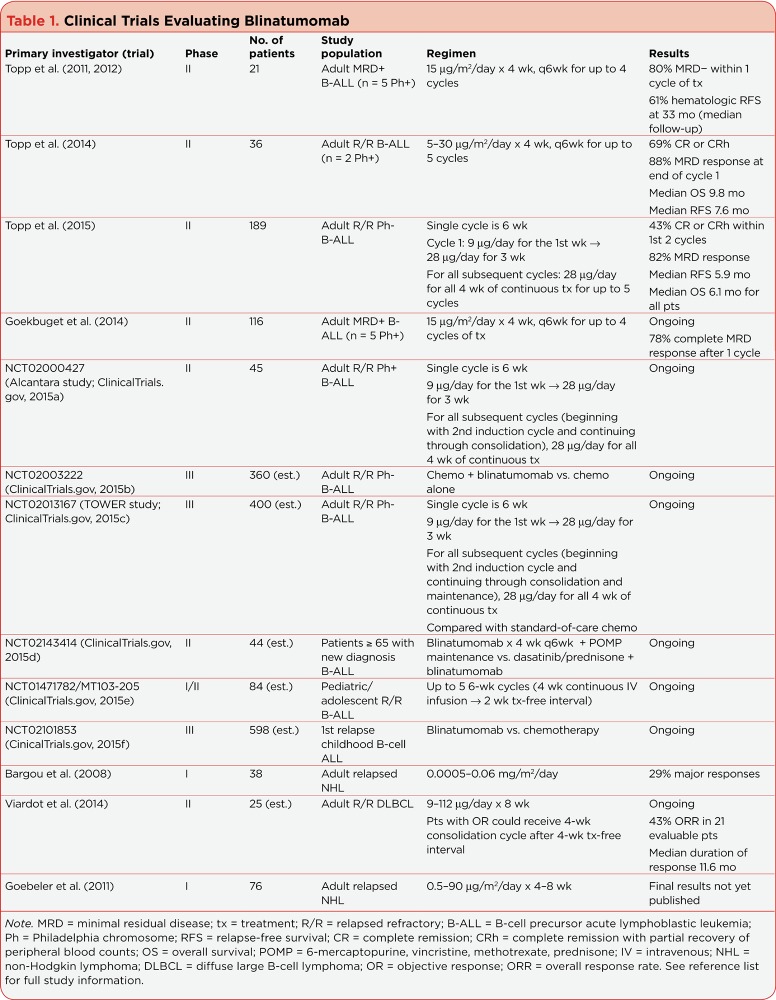
Clinical Trials Evaluating Blinatumomab

Blinatumomab received accelerated US Food and Drug Administration (FDA) approval in December 2014 for the treatment of Ph– relapsed or refractory B-ALL (not T-cell ALL), based on the results of a multicenter, international, open-label, single-arm, phase II clinical trial (Topp et al., 2015). Patients with Ph– B-ALL who were primary refractory after induction therapy, relapsed within 12 months of first remission or receiving allogeneic HSCT, or failed to respond to or relapsed after one or more salvage therapies were included in this study.

Blinatumomab was administered as a continuous intravenous infusion in 4-week cycles, followed by 2 treatment-free weeks, for up to 5 cycles. To prevent cytokine release syndrome (CRS), dosing was stepwise, with 9 µg/day for 1 week, followed by 28 µg/day for 3 weeks during cycle 1. Additionally, dexamethasone was administered up to 3 days prior to the infusion, for a maximum of 5 days, in patients with bone marrow blasts greater than 50%, peripheral blood blasts greater than 15,000 cells/µL, or an elevated lactate dehydrogenase (LDH) level in an effort to prevent CRS. All patients were premedicated with dexamethasone 20 mg within 1 hour of each treatment initiation and prior to the dose step in cycle 1. Patients could receive an allogeneic HSCT at any time during the clinical trial.

Of 189 patients treated and included in the analysis, 34% had relapsed after an allogeneic HSCT, and 51% had received at least one salvage regimen but no prior allogeneic HSCT. Patients received blinatumomab for a median of 42.2 days, and 51% of patients received dexamethasone in the pretreatment phase secondary to high tumor burden.

The primary endpoint of the study was CR, defined as ≤ 5% bone marrow blasts, platelets > 100,000 cells/µL, absolute neutrophil count (ANC) > 1,000 cells/µL, or CR with partial hematologic recovery (CRh), defined as bone marrow blasts, platelets > 50,000 cells/µL, and ANC > 500 cells/µL.

Within the first two cycles, 43% (95% confidence interval [CI] = 36%–50%) of patients achieved a CR or CRh. More patients with less than 50% bone marrow blasts at baseline had a CR or CRh compared with those patients with 50% or more blasts at baseline (73%; 95% CI = 60%–84% vs 29%; 95% CI = 22%–38%). A total of 45% of patients who had achieved CR or CRh were alive and in remission at a median follow-up of 8.9 months. Median relapse-free survival was 5.9 months for patients who achieved CR or CRh, and OS for all patients was 6.1 months. History of a prior allogeneic HSCT did not impact CR or CRh within the first two cycles of blinatumomab (45%, allogeneic HSCT vs 42%, no allogeneic HSCT).

Minimal residual disease (MRD) was evaluated in 73 patients who achieved CR or CRh, with 82% achieving MRD negativity. Median OS for MRD responders was improved compared with for MRD nonresponders (11.5 months; 95% CI = 8.5 to not estimable vs. 6.7 months; 95% CI = 2.0 to not estimable).

## ADVERSE EFFECTS

The most common adverse reactions (≥ 20%) reported in the multicenter, international, open-label, single-arm, phase II clinical trial are highlighted in Table 2 (Topp et al., 2015). The incidence of grade 3/4 adverse reactions was reported in 38%/30%, respectively, and the reactions were most commonly febrile neutropenia, anemia, and neutropenia (Topp et al., 2015).

**Table 2:**
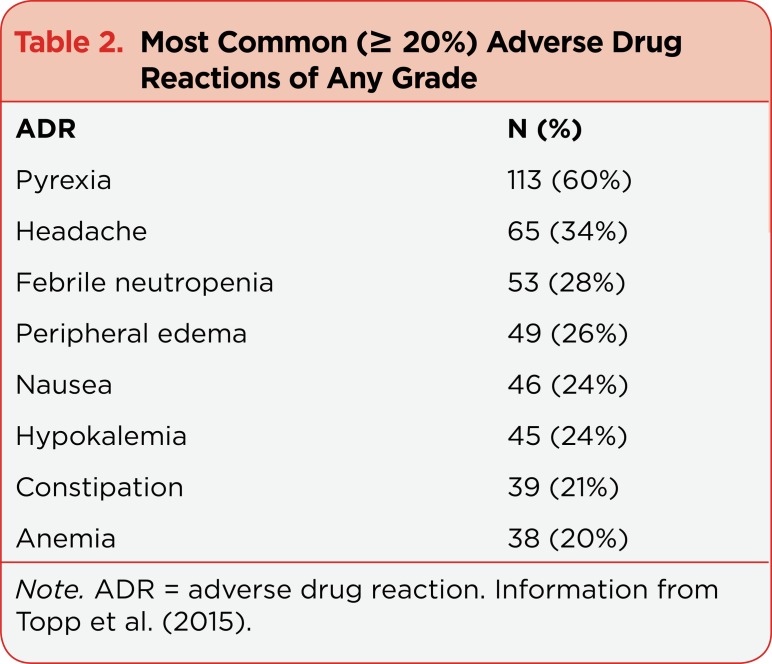
Most Common (≥ 20%) Adverse Drug Reactions of Any Grade

Dose reductions were needed in 10% of patients, and 18% discontinued treatment permanently due to adverse events (Topp et al., 2015). Of the population, 15% experienced a fatal adverse event, the majority attributed to infection (Topp et al., 2015), and none was reported in patients who had achieved remission.

Blinatumomab has black box warnings for CRS and neurologic toxicities. In this phase II study, three patients (2%) experienced grade 3 CRS, defined according to the National Cancer Institute Common Terminology Criteria for Adverse Events (NCI-CTCAE) version 4.0 (Topp et al., 2015). Signs and symptoms associated with CRS include, but may not be limited to, fever, asthenia, headache, nausea, transaminitis, hyperbilirubinemia, and hypotension (Amgen, 2014).

Due to immune system activation and T-cell expansion, increased levels of cytokines have been observed within the first 2 days after the start of blinatumomab (Amgen, 2014). In this phase II study, CRS was uncommon and less severe in comparison to a previous single-center phase II study (Topp et al., 2014), likely secondary to the recommended dose escalation as well as the prephase dexamethasone therapy required in patients with a high tumor burden (Topp et al., 2015). Infusion reactions may present in a similar manner as CRS and may be mitigated by premedicating with dexamethasone.

Neurologic toxicities of any grade were reported in 52% of patients in the phase II study (Topp et al., 2015). The majority of the events were grade 1/2 (76%), and the events most commonly reported (≥ 5%) as tremor, dizziness, confusion, encephalopathy, ataxia, and somnolence. These events occurred early on in therapy, with 87% reported in cycle 1, and a median time to onset was 7 days (Amgen, 2014). Grade 3 and 4 neurologic toxicities were reported in 11% and 2% of patients, respectively. Table 3 highlights the most common grade 3/4 neurologic toxicities reported.

**Table 3:**
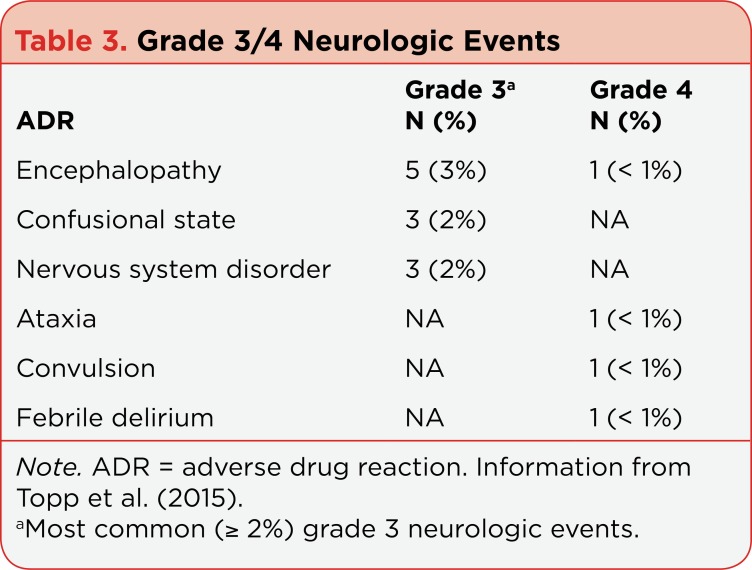
Grade 3/4 Neurologic Events

## DOSING AND ADMINISTRATION

Blinatumomab is given as a flat-dose continuous infusion for 28 straight days followed by a 2-week treatment-free period. In cycle 1, patients are given 9 µg/day for 7 days; if that is tolerated, the dose is escalated to 28 µg/day thereafter. All future cycles consist of the 28 µg/day dosing only. Hospitalization with close monitoring of infusion reactions and CRS is recommended for at least the first 9 days of cycle 1 and the first 2 days of cycle 2. If the continuous infusion is withheld for more than 4 consecutive hours for any reason, reinitiation should occur under close medical supervision or within a hospital. As a preventive measure, dexamethasone at 20 mg should be given 1 hour prior to the first dose of each cycle, prior to dose escalation on day 8 of cycle 1, and prior to re-initiation if the infusion is held for more than 4 hours.

Dosing adjustments and alterations of the infusion schedule are recommended based upon the grading of toxicities using the NCI-CTCAE. For any grade 3 toxicity, the infusion of blinatumomab should be held until the toxicity resolves to grade 1 or lower. Once the infusion is restarted, the 9-µg/day dosing should be used for the first 7 days; if that is tolerated, the infusion can be reescalated to 28 µg/day. If toxicity recurs at the 9-µg/day dosing level, blinatumomab should be discontinued permanently. In addition, if blinatumomab results in more than one seizure or any grade 4 toxicity, it should be discontinued permanently.

The administration of blinatumomab should occur through a dedicated lumen, and the line should never be flushed, even when infusion bags are changed. The doses should be delivered at a constant flow rate through an infusion pump. The infusion bags may be prepared to last either 24 hours (1 dose) or 48 hours (2 doses). The concentration of these bags will vary, but not the volume. Therefore, the recommended continuous infusion rates are 10 mL/hr and 5 mL/hr, respectively. At the time of bag change (24 or 48 hours from initiation), any unused solution in the intravenous line or infusion bag should be discarded per institutional policy (Amgen, 2014).

## PRACTICE IMPLICATIONS

Blinatumomab is a complex therapy, due to its properties and mechanism of action as well as the logistics involved with drug preparation, administration, and management of toxicities. It is imperative for patients to receive therapy under the care of health-care professionals (HCPs) who have experience in the use of this drug, with resources for close patient monitoring.

The FDA has required blinatumomab to have a Risk Evaluation and Mitigation Strategy (REMS) program. The program is in place to inform HCPs about the serious risks associated with blinatumomab, including CRS, neurologic toxicities, and preparation and administration errors. Within 30 days of the REMS approval date, Amgen sent an REMS letter to HCPs, hospital and home health-care pharmacists, and members of professional societies, who are likely to prescribe or dispense blinatumomab. Amgen will repeat the communication every 6 months for 18 months. At 18 months, 3 years, and 7 years from the initial REMS approval date, Amgen will submit REMS assessments to the FDA.

Institutions administering blinatumomab should develop an algorithm for the management of potentially severe adverse effects associated with this agent, including CRS and neurologic toxicities. A multidisciplinary approach is needed for the development of institution-specific algorithms. This approach should involve all team members who care for the patient, including nurses, pharmacists, and physician representatives.

Supportive care measures should be used to manage CRS, and depending on the severity, infusion interruption, corticosteroids, and/or tocilizumab (Actemra) administration may be necessary (Lee et al., 2014). Patients should also be monitored closely for neurologic toxicities. Depending on the grade of severity, supportive care measures, in addition to infusion interruption, and the administration of dexamethasone may be necessary to manage neurologic events.

It is important to note that the multicenter, international, open-label, single-arm, phase II clinical trial conducted by Topp et al. excluded patients with a history or presence of clinically relevant central nervous system (CNS) pathology, including epilepsy, seizure, paresis, aphasia, psychosis, organic brain syndrome, dementia, Parkinson’s disease, cerebellar disease, stroke, or severe brain injuries (Topp et al., 2015). Patients with active CNS involvement of their ALL were also excluded (Topp et al., 2015). Therefore, there are limited data available in this patient population.

Patients are required to be hospitalized for a portion of the cycle. The length of hospitalization beyond what is required will be dependent upon the patient’s safety and tolerability of blinatumomab. Therefore, transition of care from inpatient to outpatient settings for continuation of therapy may occur. This step requires careful coordination with home infusion and/or outpatient infusion centers that are trained and qualified to prepare and administer blinatumomab.

The cost of blinatumomab is a potential barrier for patients to receive this efficacious therapy. Per cycle, the cost of blinatumomab is approximately $106,800 (average wholesale price, $3,814.28 per 35-µg vial; Truven Health Analytics, 2015). This price does not include other costs that may be associated with the therapy, such as hospitalization, drug preparation, drug administration, infusion pump and supplies, and management of toxicities. Patients can receive up to two induction cycles, before CR or CRh might be achieved, increasing the potential drug cost to approximately $213,600. In patients who achieve CR or CRh, three additional cycles of consolidation treatment can be administered, for a total of up to five cycles, further increasing the cost. The drug cost for blinatumomab makes it one of the most expensive oncology drugs available (Nelson, 2014).

## SUMMARY

Up to 50% of adult patients with ALL will relapse with chemoresistant disease (Gokbuget & Hoelzer, 2009). Treatment options in this setting are limited, making the development of therapies with alternative mechanisms of action necessary. Blinatumomab has demonstrated impressive CR rates in relapsed or refractory Ph– B-ALL, including heavily pretreated patients, leading to its FDA approval for this indication. It has also been incorporated into the NCCN guidelines as a therapeutic option in this setting (NCCN, 2014).

Blinatumomab has been associated with severe and life-threatening reactions, including CRS and neurologic toxicities. Thus, it is prudent to monitor patients closely for the development of these toxicities. The administration and cost of the agent could present challenges to its widespread use. Ongoing research is needed to further define other populations that may also benefit from this therapy.
